# Suicide Risk in Military Personnel during the COVID-19 Health Emergency in a Peruvian Region: A Cross-Sectional Study

**DOI:** 10.3390/ijerph192013502

**Published:** 2022-10-19

**Authors:** Mario J. Valladares-Garrido, Cinthia Karina Picón-Reátegui, J. Pierre Zila-Velasque, Pamela Grados-Espinoza, Cristian M. Hinostroza-Zarate, Virgilio E. Failoc-Rojas, César Johan Pereira-Victorio

**Affiliations:** 1 South American Center for Education and Research in Public Health, Universidad Norbert Wiener, Lima 02002, Peru; 2 Epidemiology Office, Hospital Regional Lambayeque, Chiclayo 14000, Peru; 3 Facultad de Medicina Humana, Universidad de San Martín de Porres, Chiclayo 14000, Peru; 4 Facultad de Medicina Humana, Universidad Nacional Daniel Alcides Carrion, Pasco 19001, Peru; 5 Red Latinoamericana de Medicina en la Altitud e Investigación (REDLAMAI), Pasco 19001, Peru; 6 Research Unit for Generation and Synthesis Evidence in Health, Universidad San Ignacio de Loyola, Lima 02002, Peru; 7 School of Medicine, Universidad Continental, Lima 02002, Peru

**Keywords:** COVID-19, mental health, suicide risk, public health, post-traumatic stress disorder, sleep quality

## Abstract

Military personnel represent a frontline group exposed to multiple stressors. These factors have increased during the COVID-19 pandemic, predisposing to the development of suicidal risk (SR). Given the few studies conducted in this population, we evaluated the prevalence of SR and its associated factors during the health emergency. A cross-sectional survey study was conducted in person among 514 participants in Lambayeque, Peru in 2021. The outcome was SR, and the exposures were depression (PHQ-9), anxiety (GAD-7), PTSD (PCL-C), and other sociodemographic variables. The prevalence of SR was 14.0% (95% CI: 11.12–17.31%) and was significantly higher in people with a family history of mental health (PR: 2.16; 95% CI: 1.13–4.15) and in those with moderate clinical insomnia (PR: 2.21; 95% CI: 1.19–4.12). Military personnel with high resilience had a lower prevalence of SR (PR: 0.54, CI: 0.31–0.95). Anxiety was associated with a higher prevalence of SR (PR: 3.27; 95% CI: 1.76–6.10). Our findings show that at least 1 out of 10 military personnel are at risk of suicide. Special attention should be paid to the associated factors to develop interventions and reverse their consequences. These results may be useful in policy implementation and general statistics of SR in the local and regional context.

## 1. Introduction

Worldwide, one person dies by suicide every 40 s, which annually represents the cause of death of approximately 800,000 people [1]. The annual mortality rate worldwide has been estimated by the World Health Organization (WHO) at 10.7 per 100,000 people [2]. Currently, it is the second leading cause of death among young people aged 15–29 years, where most suicide deaths occur in middle and low-income countries [3]. In our country, a suicide rate between 2.31 and 6.00 has been reported [4], with a sustained increase.

It is necessary to emphasize that there are groups that deserve special evaluation for having a higher risk of suicide, such as military personnel [5]. According to one study, the mortality rate by suicide in North American military personnel has been estimated at 18.5 per 100,000 persons/year, which represents the second cause of death in this population [6], and is higher than that reported by the WHO, with estimates of lifetime prevalence of suicidal ideation, suicide plans and suicide attempts of 13.9%, 5.3% and 2.4%, respectively [5]. Likewise, an association between PTSD and suicidal ideation and attempt has been identified in this group of people [7]. Similarly, a systematic review that included military personnel, where the main exposure was a concussion or mild traumatic brain injury, identified that these conditions confer a twofold increased risk of suicide [8]. However, a series of protective factors against suicide risk have also been studied, such as programs that facilitate assistance to the military, assistance with social networks, family support and educational counseling sessions, factors that are little evaluated [9]. Thus, it is critical to evaluate this group.

However, the COVID-19 pandemic deteriorated the mental health [10,11,12,13], that according to WHO the pandemic has generated a 25% increase in the prevalence of anxiety and depression worldwide [14], particularly of various groups of professionals [15]. Among them, the military, as a group of first line of defense worldwide, supported by a study that evaluated the rates of death by suicide in different generational groups, identifying that since the beginning of the pandemic, the military has observed increases from 55% to 82% in suicide rates [16]. However, it is highlighted that data and quality on suicide attempts are low worldwide, which is related to insufficient information [2], so that in September 2021, the World Health Assembly [17], accepted indicators and the implementation of measures to achieve the reduction in suicide rates to one third by 2030 [18].

Currently it is postulated that the interpersonal theory of suicide, which contains three constructs: *perceived burden*, *frustrated belonging* and *acquired capacity* [19]. They should be developed in people with suicidal tendencies, in addition to having determinants associated with demographic parameters such as gender, low social status, dysfunctional or violent environment [20], and a history of psychiatric illness, which predisposes to the vast majority of suicides and suicide attempts, where they are 10 times higher than in the general population [21]. This result is supported by studies that identify a relationship between depression, post-traumatic stress disorder (PTSD) and suicidal ideation [22,23].

However, reports of suicide risk in military personnel present several limitations. First, the data reported so far on suicide risk in times of pandemic is scarce; however, an increase in its prevalence is expected [24]; second, the use of a retrospective methodology is based on secondary data, such as the use of death certificates that depend on forensic physicians and therefore there is variability in the way of their evaluation and results [5]; third, the studies do not report a probabilistic type of sampling [5,25]; fourth, according to a systematic review there is a lack of consistency in the multivariate results, and analytical approaches on methodological differences such as the use of a diagnostic interview versus electronic medical record, and important associated factors are not synthesized [7], such as having children, working time, family history of mental health, presence of insomnia, resilience and mental health outcomes, anxiety, depression.

The factors mentioned above have been shown to have a strong influence on the development of suicide risk [26]. For example, in Ethiopian psychiatric patients, those who had family members with a history of mental illness were 3.03 times more likely to have suicidal ideation [27]. In active U.S. military, subjects with insomnia symptoms were three times more likely to report suicidal ideation [28]. In the general population in Taiwan, the risk of suicide attempts among patients with insomnia was 3.5 times higher compared to those without insomnia [29]. There is also evidence that disrupted sleep is a risk factor for suicide, and that nighttime wakefulness and severity of insomnia increase the likelihood of suicidal ideation [30]. Notably, resilience has been shown to become a measure of active coping in the pandemic [31]. A meta-analysis reported that anxiety was found to be a statistically significant predictor of suicidal ideation (OR = 1.49; 95% CI: 1.18–1.88) and suicide attempts (OR = 1.64; 95% CI: 1.47–1.83) [32]. Finally, a study showed that the risk of suicide in U.S. veterans was higher for those who suffered from depression [33].

In relation to Latin American studies, the scarcity of research in this area stands out, and those that are found have limitations in the type of sampling used and the sample size [25,34]. Therefore, the present study has as general objective to identify the prevalence of suicide risk in military personnel in a region of Peru, and among the specific objectives, to identify the factors associated with suicide risk such as sociodemographic, mental health, the presence of insomnia and the level of resilience.

## 2. Materials and Methods

### 2.1. Study Design, Population, and Sample

A cross-sectional study was conducted using data from a previous study that identified factors associated with post-traumatic stress disorder among 820 military personnel. The study population worked in line-of-defense activities during the COVID-19 pandemic in Lambayeque, northern Peru. This region was severely affected by the health emergency in the first two pandemic waves [35]. Approximately 1400 military personnel were actively working during the pandemic period at the time of the primary study [36]. A sample size of 485 military personnel was estimated in the primary study, using a 12.8% expected proportion, 99% confidence level, 2.5% precision. Because of the large sample size, a significance level of 0.01 was set to test the primary hypothesis. To this, we added a 10% rejection rate and 10% for incomplete questionnaires in the variables of interest, resulting in a final sample of 582 military personnel. Using an expected prevalence of suicidal ideation of 24.6% reported in Colombia [25] and the observed prevalence of suicidal ideation of 14% in this investigation, we estimated a statistical power of 100%. The inclusion criteria for the primary study were that the personnel were actively working during the pandemic and had at least 1 month of work. In this study, we included only the completed and valid questionnaires of the Plutchik suicide risk scale. We excluded 196 records, resulting in a final sample size of 514 for this analysis. The sampling type applied was non-probabilistic by snowball.

### 2.2. Procedure

The enrollment of participants began with a request for authorization from the Lambayeque military brigade to conduct interviews with its active members. The interviews were conducted in person with structured questionnaires conducted by the field interviewers, in three groups, in two different shifts (morning and afternoon), with an approximate duration of 2 h. These interviews were conducted under strict respect for the biosecurity measures implemented in the military center.

The data were collected and managed using the Research Electronic Data Capture system (REDCap). REDCap is a secure online platform for designing, managing, entering, and rigorously capturing surveys and online databases for research [37,38]. To design the online survey, a template was created in which all the data collection forms were to be included. We clicked on “Add new instrument” and created 2 forms: (1) informed consent and (2) data collection questionnaires. This process was performed within the Online Designer tool.

Then, we used the Survey Queue tool. This tool allowed us to combine the list of all questionnaires into one single form for each participant. To combine all questionnaires into this single form, we activated the Survey Queue in the REDCap project, we then navigated to “Online Designer”, and clicked on the Survey Queue icon located above the data collection instruments. Immediately, a “Set up Survey Queue” box appeared. Next, we clicked the Enable icon for each questionnaire we wanted to set up. Under the “Show survey in survey queue when…” column, we used the drop-down menu to indicate when each questionnaire should be shown to the participant. We used the Branching Logic tool in the Survey Queue to display the questions in the questionnaires. The Branching Logic tool allowed to display the questionnaires to the participants compiled on a single form automatically, only if the participant provided informed consent.

In addition, we used other tools in the REDCap project to ensure the correct arrangement, provision, and completion of questionnaires: unique and anonymized identifiers on each form, questionnaires ordered in a consistent way [(1) informed consent, (2) general data, and (3) quantitative scales], use of conditional logic for skip questions, mandatory fields in questions to avoid missing, minimum, and maximum ranges in numerical variables, and use of groups matrix tool for Likert scale responses. Finally, a public survey link was created using the Manage Survey Participants tool. Before starting the study, the survey link and form were verified to work correctly.

### 2.3. Questionnaire

It consisted of 9 sections covering (1) Sociodemographic data; (2) Suicide Risk Scale; (3) Generalized Anxiety Disorder Scale (GAD-7); (4) Depression Scale (PHQ-9); (5) Posttraumatic Stress Disorder Questionnaire (PCL-C); (6) Burnout Syndrome Maslach Burnout Inventory; (7) Insomnia Questionnaire (ISI); (8) Fear of COVID-19 Scale; (9) Physical Activity Questionnaire (IPAQ-S).

In the general data, information was obtained on age in years, sex (male, female), marital status single (no, yes), religion (none, Catholic, non-Catholic), previous pathologies (hypertension, diabetes), report of frequent alcohol and tobacco consumption, self-reported weight and height, previous personal and family history of mental illness, mental health support during the pandemic, having confidence in the government to handle the pandemic, and time working in the face of the COVID-19 pandemic at military headquarters (1 to 6 months, 7 to 12 months, 13 to 18 months, 19 months or more).

### 2.4. Outcome

Plutchik suicide risk scale: This is a 15-question, self-administered, yes/no questionnaire. Each affirmative answer scores 1 and the sum of the scores equal to or greater than 6 indicates the presence of suicidal risk [39]. It has demonstrated a sensitivity and specificity of over 68%, and has been validated in the civilian population and in members of the public order [40]. Its reliability through Cronbach’s Alpha 80, which has also been validated for the Latin American population [41]. In this research, we obtained high overall internal consistency (Cronbach’s alpha: 0.94) and for each item (Cronbach’s alpha > 0.93).

### 2.5. Exposures

Resilience: to assess resilience we used the Connor-Davidson short resilience scale (CD-RISC) which consists of 10 items that can be used as a reliable and valid measure of resilience. The original version has good properties, with a Cronbach’s alpha of 0.89 (general population) and a test–retest reliability of 0.87 (people with generalized anxiety disorder (GAD) and posttraumatic stress disorder (PTSD) [42]. It was assessed through a Likert scale with 5 options by scoring from 0–4. We used the cut-off point of 30 to categorize high (>30), and low resilience (<30) [43]. In general, it shows excellent psychometric properties and allows an efficient measurement of resilience [44]. For this study, the Cronbach’s alpha coefficient was 0.97.

Anxiety: the GAD-7 questionnaire is a self-administered unidimensional scale designed to assess the presence of GAD symptoms. A cut-off point was identified that optimized sensitivity (89%) and specificity (82%) [45]. It consists of 7 items where scores range from 0 (not at all) and 3 (almost every day). Thus, the total score ranges from 0 to 21. Reliability (internal consistency) was high; Cronbach’s alpha = 0.875 [46]. For this study, the Cronbach’s alpha coefficient was 0.93.

Depression: the PHQ-9 depression scale was used; this is a psychometrically reliable instrument for the diagnosis of depression, and easy to use in the context of the primary care system in Peru [47]. It consists of 9 items that evaluate the presence of depressive symptoms (corresponding to DSM-IV criteria) present in the last 2 weeks. Each item has a severity index corresponding to: 0 = “never”, 1 = “some days”, 2 = “more than half of the days” and 3 = “almost every day”. It presents an acceptable internal consistency with a Cronbach’s Alpha coefficient of 0.835. Additionally, optimal sensitivity (88%) and specificity (92%) values [48]. For this study, the Cronbach’s alpha coefficient was 0.92.

Post-traumatic stress disorder (PCL-C): includes 17 items, which correspond to the set of symptoms identified in the DSM-IV-TR for criteria B, C and D (intrusive re-experiencing, avoidance, and activation, respectively). In the instructions on the instrument, you are asked to indicate how much “bother” each of the 17 symptoms has caused you during the past month, using a Likert scale, where 1 equals “no” bother, 2 “a little”, 3 “moderately”, 4 “a lot” and 5 “too much”. The minimum total score of the instrument is 17 and the maximum score is 85. According to the original version, a score equal to or greater than 44 indicates the presence of PTSD symptoms or “possible case” [49], the instrument showed high internal consistency (α = 0.94) and adequate test–retest reliability (r = 0.82) [50]. For this study, the Cronbach’s alpha coefficient was 0.95.

Maslach Burnout Inventory Human Services Survey (MBI-HSS): it consists of 22 items, it is distributed in three scales named, Emotional Exhaustion (9 items), Personal Accomplishment at Work (9 items), and Depersonalization (5 items). The reliability values of the scales also show high internal consistency (α = 0.882) [51]. For its identification in Peruvian health personnel, it is recommended to use cut-off points predetermined by the creator of the instrument AE > 26, DP > 9 RP < 34) [52]. For this study, the Cronbach’s alpha coefficient was 0.91.

Insomnia Questionnaire (ISI): It is composed of 7 items that assess the nature, severity, and impact of insomnia. Higher scores reflect a greater degree of insomnia [53]. Cronbach’s alpha was 0.82. It has been validated in older adults, primary care patients, and the general Spanish-speaking population [54]. For this study, the Cronbach’s alpha coefficient was 0.88.

Physical Activity Questionnaire (IPAQ-S): An instrument that considers the four components of physical activity (leisure time, home maintenance, occupational, and transportation) [55], consists of 9 items and assesses the physical activity reported in the last 7 days. It allows obtaining a weighted estimate of total physical activity from the activities reported per week, to categorize physical activity as: intense, moderate, mild or inactive. It has been validated in Spanish-speaking populations and applied in Latin American population [56]. For this study, the Cronbach’s alpha coefficient was 0.64.

COVID-19 fear scale: it consists of seven items and has been shown to be reliable and valid for assessing fear of COVID-19 among the general population; with a Cronbach’s alpha of 0.82 [57]. The Spanish version of the COVID-19 Fear Scale in a sample of the Peruvian population showed adequate psychometric properties in terms of reliability and validity [58]. We defined as the presence of fear of COVID-19 with a score above 16.5 [59]. For this study, the Cronbach’s alpha coefficient was 0.94.

### 2.6. Statistical Analysis

Survey data was downloaded from REDCap as a *.csv* file and then imported and analyzed in Stata 16.1 (College Station, TX, USA: StataCorp LL).

In the descriptive analysis, we described categorical variables as absolute and relative frequencies, and numerical variables as mean (standard deviation) or median (range) values, as appropriate, after evaluation of the normal distribution assumption.

In the bivariate analysis, we used the chi-square test, after evaluation of the expected frequency assumption, to determine whether categorical variables were associated with suicide risk. In the case of numerical variables (age in years), we used the Mann–Whitney U test. We worked with a significance level of 5%.

We performed simple and multiple regression analyses to identify factors associated with suicidal risk. We estimated prevalence ratios (PR) and 95% confidence intervals (95% CI). We used generalized linear models (GLM) with Poisson distribution family, robust variance and log link function. In the multiple model, we entered the variables that were significantly associated in the simple model. We evaluated collinearity between the variables of interest.

### 2.7. Ethical Aspects

The primary study protocol was evaluated and approved by the Institutional Research Ethics Committee (CIEI) of Universidad Privada Norbert Wiener [Norbert Wiener Private University]. Informed consent was obtained from each participant, and the data were anonymous, coded, and confidential. The data collected were recorded in the data entry system (REDCap), to facilitate validation and quality control of data entry. It is worth mentioning that prior to data entry, approval of the electronic version of the informed consent form was requested.

## 3. Results

The median age was 22 years old, with an age range of 19 to 32 years old. Male gender predominated 95.7% (*n* = 492). A total of 26.5% (*n* = 136) reported having children. In relation to substance use, alcoholism and smoking were present in 17.1% (*n* = 88) and 6.8% (*n* = 35), respectively. Regarding medical history, 9.3% (*n* = 48) had hypertension, 33.8% (*n* = 171) were overweight, and 8.2% (*n* = 42) had a history of seeking mental health care. Regarding mental health outcomes, subclinical insomnia was present in 18.1% (*n* = 93), mild depression in 18.5% (*n* = 95), and PTSD in 7.2% (*n* = 37). The prevalence of suicidal risk was reported in 14.0% (*n* = 72; 95% CI: 11.12–17.31%). (Table 1).

**Figure 1:**
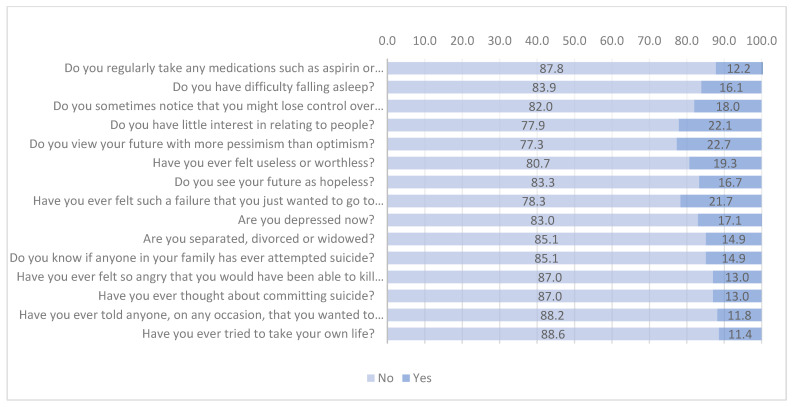
Frequency of responses according to each item of the Plutchik suicide risk scale.

According to the items of the Plutchik suicide risk scale, most of the participants responded higher on the item “Do you view your future with more pessimism than optimism?” (22.7%), followed by the items “Do you have little interest in relating to people?” (22.1%), and “Have you ever felt such a failure that you just wanted to go to bed and give it all up?” (21.7%) (Figure 1).

In the bivariate analysis (Table 2), significant differences were found in the prevalence of SR, according to being single (16.2% single vs. 7.6% no single, *p* = 0.014), having children (8.8% children vs. 9.9% no children, *p* = 0.042), working time (8.8% 19 months or more vs. 19.2% 1 to 6 months, *p* = 0.047), family history of mental health (40.9% family history vs. 12.8% no family history, *p* < 0.001), insomnia (44% severe clinical insomnia vs. 11.3% absent insomnia, *p* < 0.001), resilience (7.1% high resilience vs. 19.4% low resilience, *p* < 0.001). Likewise, with mental health outcomes, anxiety (63.6% severe anxiety vs. 8.2% no anxiety, *p* < 0.001), depression (50% severe depression vs. 8.5% minimal depression, *p* < 0.001), and PTSD (29.7% PTSD vs. 12.8% no PTSD, *p* = 0.004).

**Figure 2:**
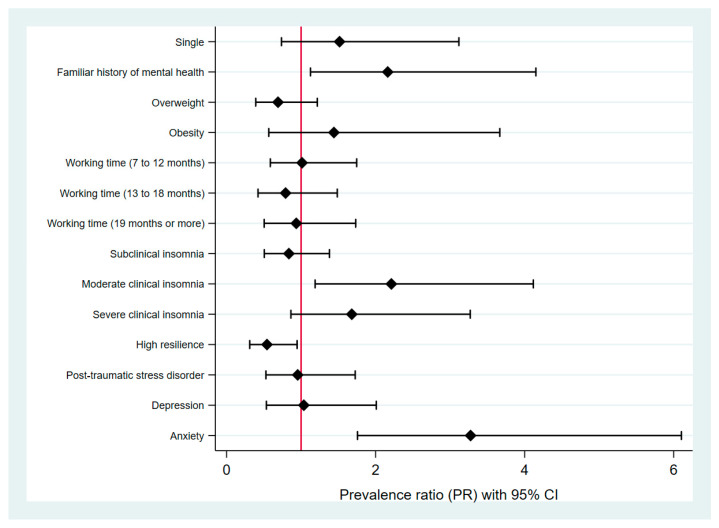
Estimates plot of the factors associated with suicidal risk in multiple regression analysis.

In the multiple regression analysis, the prevalence of SR was higher in people with a family history of mental health (PR: 2.21; 95% CI: 1.12–4.33), with moderate clinical insomnia (PR: 2.21; 95% CI: 1.19–4.12), and lower with the presence of high resilience (PR: 0.54, CI: 0.31–0.95). In relation to mental health outcomes, a positive association was found with anxiety (PR: 3.27; 95% CI: 1.76–6.10) (Figure 2).

## 4. Discussion

### 4.1. Prevalence of Suicidal Risk

The prevalence of suicidal risk was 14%. This is similar to that reported by Mateo K. et al. and Robert J. et al. in the “Army STARSS” study conducted in the United States, in 5428 non-deployed active soldiers, survey collected over 8 months and 38,237 new soldiers, that were collected in approximately 2 years, reporting a prevalence of suicidal ideation of 13.9% [5], and 14.1% [60], respectively. Both studies shared sociodemographic variables such as age, gender, marital status, ethnicity, marital status; both studies used a modified self-report/baseline version of the Columbia Suicide Severity Rating Scale (C-SSRS).

In Colombia, Alvaran L. et al. reported a prevalence of suicidal ideation of 24.6% in 410 soldiers attached to a battalion [25]. However, this differs from the findings of Kopacz et al. in the study conducted in the United States in 472 veterans, mostly from Vietnam, in which a prevalence of 71% [7,61]; this result is attributed to the fact that this population is known to have an increased risk of suicide and that factors related to spirituality were shown to be associated with the prediction of suicidal ideation [61]. In turn, Roberge et al. conducted a study in 290 U.S. veterans, where 46% reported low risk and 10% high risk of suicide; this finding could be explained by the fact that the population at the time of the study was participating in a cognitive processing therapy, the sample was smaller and was conducted before the pandemic [62]. In Huancayo, Peru; where 84% reported a low risk, 10% moderate risk and 6% high suicide risk in 200 soldiers in voluntary military service, the population being entirely male and with a median age of 19 years, in which these findings could be explained due to the limited number of the sample and that it was conducted pre-pandemic [34].

### 4.2. Factors Associated with Suicidal Risk

In our research, we found that having a family member with a mental health problem increased the prevalence of suicidal risk by 116%. This is similar to what was found in Ethiopian psychiatric patients, where those who had family members with a history of mental illness were 3.03 times more likely to have suicidal ideation than those who did not have family members with a history [27]. It is also similar to another study, conducted in Eritrean refugees, who reported three times higher risk of reporting suicide attempt relative to those who did not have relatives with mental disorder [63]. Our finding could be explained by the fact that in these families there could be a form of learning; if one family member attempted suicide, another young family member could adopt this model of solution to emotional difficulties or coping with distress [64]. For this reason, it would be very important for military personnel to have their personal and family medical history reviewed upon entering the institution in order to detect risk factors for suicide [65] and to receive psychological counseling from their institution.

Having moderate clinical insomnia increases 121% the prevalence of suicidal risk. This is similar to findings in active U.S. military, where subjects with insomnia symptoms were three times more likely to report any suicidal ideation [28]. It is also similar to another study in the general population in Taiwan, where the risk of suicide attempts among patients with insomnia was 3.5 times higher compared to those without insomnia [29]. Our finding could be explained by physiological and psychological mechanisms: the physiological mechanism includes a reduction in serotonin and a dysfunction of the hypothalamic-pituitary-adrenal axis; and the psychological mechanism is associated with dysfunctional beliefs and attitudes about sleep [29]. In addition, military personnel must perform night watches so they must stay awake at night or interrupt their sleeping hours. Therefore, being awake at night and the associated hypofrontality that occurs during the night and/or with sleep loss may be another mechanism by which insomnia increases the risk of suicidal ideation [28]. Likewise, there is evidence that disrupted sleep is a risk factor for suicide, and that nighttime wakefulness and severity of insomnia increase the likelihood of suicidal ideation [30]. As already mentioned, most military personnel do not have fixed work schedules and this influences their sleep; therefore, it is recommended that sleep programs be conducted in military personnel who are found to have insomnia problems at least semi-annually.

Military personnel with a high resilience pattern reduce the prevalence of suicidal risk by 46%. This could be due to the fact that in our study the majority of participants were young; and being young is one of the basic determinants of resilience, as is having lower rates of psychiatric disorders [66]. Furthermore, resilience has been shown to become a measure of active coping in the pandemic [31]. Our result is similar to that reported by a study that identified a low prevalence of disorders in Special Operations Command in soldiers who demonstrated hyperresilience [67]. These findings support that resilience plays an important role in conveying the impact of uncertainty on suicidal ideation and that resilient individuals are better equipped to cope with difficult times [68].

Having anxiety significantly increases the prevalence of suicidal risk. This is similar to that reported by Conner et al. who found that US military members with anxiety were associated with an increased risk of suicide [69]. US veterans with anxiety were also found to have a 3-fold increased risk of suicidal ideation [70]. This finding is also supported by the meta-analysis of a systematic review, where anxiety was found to be a statistically significant predictor of suicidal ideation (OR = 1.49; 95% CI: 1.18–1.88) and suicide attempts (OR = 1.64; 95% CI: 1.47–1.83) [32]. Therefore, our finding could be explained because anxiety in the military may be exacerbated by stresses to adapt to a unique community life, exposure, discipline, and the stresses associated with ranks and combat situations [71].

Having depression positively increases the prevalence of suicidal risk in the simple model; however, in the final model that association is diluted. However, a study was found that differs from the findings, which showed that the risk of suicide in U.S. veterans was higher for those who suffered from depression [33]. This finding could be explained because the military have certain activities or characteristics during the performance of their profession such as living in operational conditions, being in multiple combat missions, having environmental restrictions and being away from the family. Therefore, due to this type of lifestyle, burnout, work stress and mental disorders such as depression and suicide are very common among them [72]. Although our study provides evidence to the contrary, further studies should be conducted.

We recommend that special attention should be paid to factors associated with the development of suicidal risk in military personnel, among the most important of which are the presence of mental health outcomes, family history of mental health, and moderate clinical insomnia. Additionally, it should be noted that military personnel in their profession are often prone to hide their personal feelings or distort their responses in order to “look good” to others, i.e., they choose responses that create a favorable impression [73]. Therefore, it is very relevant to execute measures to address this situation through the primary care health centers of the military institution with the implementation of the following measures: (a) Educate professionals about the risks of suicidal thoughts and behaviors, (b) Provide screening of patients to identify suicide risk and/or mood disturbances (anxiety and depression), (c) Use evidence-based interventions, including collaborative and multidisciplinary teams, to manage depression, and (d) Assess the presence of suicide risk factors and manage suicide risk when symptoms emerge [65].

### 4.3. Limitations and Strengths

In relation to the limitations, the cross-sectional study design does not allow us to identify causal relationships between the study variables, but as a strength, validated instruments were used in our context. Another limitation was that it was not possible to reproduce the results adequately to extrapolate them to other population groups, but as a strength it was possible to obtain a large sample. Nor was it possible to measure other variables that influence suicidal risk in the military population, such as socioeconomic level, type of family relationship, place of birth and origin [74], level of self-esteem, social skills, social support, and impulse control [75]. Finally, a possible limitation for participants not being able to participate fully is attributed to the large study protocol; however, the study encompassed several variables that could have been used in the study.

## 5. Conclusions

At least 1 in 10 military personnel are at suicidal risk. Special attention should be paid to the associated factors, such as the presence of a family member with a history of mental illness, insomnia, anxiety, depression, and resilience, to develop effective interventions that prevent mental disorders and further suicide. We recommend that health programs within the military be developed. For example, there could be workshops that screen for potential patients with mental disorders, and others that implement sleep hygiene and resilience training. We emphasize that these measures could help the military members to have a balanced mental state and wellbeing. Therefore, our results may be useful in the implementation of policies and general statistics to decrease the impact of the COVID-19 in this population group.

## Figures and Tables

**Table 1:** Characteristics of military personnel (*n* = 514).

Characteristics		*n* (%)
Age (years) *		22 (19–32)
Gender		
	Female	22 (4.3)
	Male	492 (95.7)
Single		
	No	132 (25.7)
	Yes	382 (74.3)
Religion		
	None	79 (15.4)
	Catholic	354 (68.9)
	Non-Catholic	81 (15.8)
Children		136 (26.5)
Alcoholism		88 (17.1)
Smoking		35 (6.8)
Comorbidity		
	Hypertension	48 (9.3)
	Diabetes	9 (1.8)
BMI (categorized)		
	Underweight/Normal	304 (60.1)
	Overweight	171 (33.8)
	Obesity	31 (6.1)
Personal mental health history		
	No	508 (98.8)
	Yes	6 (1.2)
Family mental health history		
	No	492 (95.7)
	Yes	22 (4.3)
Seeking mental health help		
	No	472 (91.8)
	Yes	42 (8.2)
Trust in government to handle COVID-19		
	Yes	283 (55.1)
	No	231 (44.9)
Time of work **		
	1 to 6 months	130 (26.0)
	7 to 12 months	81 (16.2)
	13 to 18 months	108 (21.6)
	19 months or more	182 (36.3)
Insomnia		
	Absence of clinical insomnia	400 (77.8)
	Subclinical insomnia	93 (18.1)
	Moderate clinical insomnia	12 (2.3)
	Severe clinical insomnia	9 (1.8)
Food insecurity		
	No	262 (51.0)
	Yes	252 (49.0)
Physical activity		
	Low	63 (12.3)
	Moderate	37 (7.2)
	High	414 (80.5)
Resilience		
	Low	288 (56.0)
	High	226 (44.0)
Fear scale		
	No	416 (80.9)
	Yes	98 (19.1)
Burnout Syndrome		
	No	464 (90.3)
	Yes	50 (9.7)
Anxiety		
	No	404 (78.6)
	Mild	74 (14.4)
	Moderate	25 (4.9)
	Severe	11 (2.1)
Depression		
	Minimal	366 (71.2)
	Mild	95 (18.5)
	Moderate	37 (7.2)
	Moderate-severe	10 (2.0)
	Severe	6 (1.2)
Post-traumatic stress disorder		
	No	477 (92.8)
	Yes	37 (7.2)
Suicidal risk		
	No	442 (86.0)
	Yes	72 (14.0)

* Median (25th percentile–75th percentile). ** Missing values.

**Table 2:** Characteristics associated with suicidal risk in military personnel.

Variables		Suicidal Risk		*p* *
		No (*n* = 442)	Yes (*n* = 72)	
		*n* (%)	*n* (%)	
Age (years) **		22 (19–32)	21.5 (19–28.5)	0.358 ***
Gender				0.497
	Female	20 (90.9)	2 (9.1)	
	Male	422 (85.8)	70 (14.2)	
Single				0.014
	No	122 (92.4)	10 (7.6)	
	Yes	320 (83.8)	62 (16.2)	
Religion				0.152
	None	63 (79.8)	16 (20.3)	
	Catholic	306 (86.4)	48 (13.6)	
	Non-Catholic	73 (90.1)	8 (9.9)	
Children		124 (91.2)	12 (8.8)	0.042
Alcoholism		78 (88.6)	10 (11.4)	0.432
Smoking		30 (85.7)	5 (14.3)	0.961
Comorbidity				
	Hypertension	40 (83.3)	8 (16.7)	0.577
	Diabetes	7 (77.8)	2 (22.2)	0.474
BMI (categorized)				0.051
	Underweight/Normal	253 (83.2)	51 (16.8)	
	Overweight	156 (91.2)	15 (8.8)	
	Obesity	26 (83.9)	5 (16.1)	
Personal mental health history				0.170
	No	438 (86.2)	70 (13.8)	
	Yes	4 (66.7)	2 (33.3)	
Family mental health history				<0.001
	No	429 (87.2)	63 (12.8)	
	Yes	13 (59.1)	9 (40.9)	
Seeking mental health help				0.957
	No	406 (86.0)	66 (14.0)	
	Yes	36 (85.7)	6 (14.3)	
Trust in government to handle COVID-19				0.927
	Yes	243 (85.9)	40 (14.1)	
	No	199 (86.2)	32 (13.9)	
Time of work				0.047
	1 to 6 months	105 (80.8)	25 (19.2)	
	7 to 12 months	67 (82.7)	14 (17.3)	
	13 to 18 months	94 (87.0)	14 (13.0)	
	19 months or more	166 (91.2)	16 (8.8)	
Insomnia				<0.001
	Absence of clinical insomnia	355 (88.8)	45 (11.3)	
	Subclinical insomnia	76 (81.7)	17 (18.3)	
	Moderate clinical insomnia	6 (50.0)	6 (50.0)	
	Severe clinical insomnia	5 (55.6)	4 (44.4)	
Food insecurity				0.666
	No	227 (86.6)	35 (13.4)	
	Yes	215 (85.3)	37 (14.7)	
Physical activity				0.375
	Low	54 (85.7)	9 (14.3)	
	Moderate	29 (78.4)	8 (21.6)	
	High	359 (86.7)	55 (13.3)	
Resilience				<0.001
	Low	232 (80.6)	56 (19.4)	
	High	210 (92.9)	16 (7.1)	
Fear scale				0.167
	No	362 (87.0)	54 (13.0)	
	Yes	80 (81.6)	18 (18.4)	
Burnout Syndrome				0.086
	No	395 (85.1)	69 (14.9)	
	Yes	47 (94.0)	3 (6.0)	
Anxiety				<0.001
	No	371 (91.8)	33 (8.2)	
	Mild	49 (66.2)	25 (33.8)	
	Moderate	18 (72.0)	7 (28.0)	
	Severe	4 (36.4)	7 (63.6)	
Depression				<0.001
	Minimal	335 (91.5)	31 (8.5)	
	Mild	71 (74.7)	24 (25.3)	
	Moderate	28 (75.7)	9 (24.3)	
	Moderate-severe	5 (50.0)	5 (50.0)	
	Severe	3 (50.0)	3 (50.0)	
Post-traumatic stress disorder				0.004
	No	416 (87.2)	61 (12.8)	
	Yes	26 (70.3)	11 (29.7)	

* *p*-value calculated with the chi-square test. ** Median—interquartile range. *** *p*-value calculated with the Mann–Whitney U-test.

## Data Availability

The dataset generated and analyzed during the current study is not publicly available because the ethics committee has not provided permission/authorization to publicly share the data but are available from the corresponding author on reasonable request.

## References

[1] Preventing Suicide: A Global Imperative. https://www.who.int/publications-detail-redirect/9789241564779.

[2] Bachmann S. (2018). Epidemiology of Suicide and the Psychiatric Perspective. Int. J. Environ. Res. Public Health.

[3] Arensman E., Scott V., De Leo D., Pirkis J. (2020). Suicide and Suicide Prevention From a Global Perspective. Crisis.

[4] Hernández-Vásquez A., Azañedo D., Rubilar-González J., Huarez B., Grendas L. (2016). Evolución y Diferencias Regionales de La Mortalidad Por Suicidios En El Perú, 2004–2013. Rev. Peru. Med. Exp. Salud Publica.

[5] Nock M.K., Stein M.B., Heeringa S.G., Ursano R.J., Colpe L.J., Fullerton C.S., Hwang I., Naifeh J.A., Sampson N.A., Schoenbaum M. (2014). Prevalence and Correlates of Suicidal Behavior among Soldiers: Results from the Army Study to Assess Risk and Resilience in Servicemembers (Army STARRS). JAMA Psychiatry.

[6] Ocampo-Ortega R. (2020). Riesgo de suicidio en militares. Rev. Cuba. Med. Mil..

[7] Holliday R., Borges L.M., Stearns-Yoder K.A., Hoffberg A.S., Brenner L.A., Monteith L.L. (2020). Posttraumatic Stress Disorder, Suicidal Ideation, and Suicidal Self-Directed Violence Among U.S. Military Personnel and Veterans: A Systematic Review of the Literature From 2010 to 2018. Front. Psychol..

[8] Fralick M., Sy E., Hassan A., Burke M.J., Mostofsky E., Karsies T. (2019). Association of Concussion With the Risk of Suicide: A Systematic Review and Meta-Analysis. JAMA Neurol..

[9] Krause-Parello C.A., Rice M.J., Sarni S., LoFaro C., Niitsu K., McHenry-Edrington M., Blanchard K. (2019). Protective Factors for Suicide: A Multi-Tiered Veteran-Driven Community Engagement Project. J. Veterans Stud..

[10] Javier-Aliaga D.J., Quispe G., Quinteros-Zuñiga D., Adriano-Rengifo C.E., White M. (2022). Hope and Resilience Related to Fear of COVID-19 in Young People. Int. J. Environ. Res. Public Health.

[11] Fernandez-Canani M.A., Burga-Cachay S.C., Valladares-Garrido M.J. (2022). Association between Family Dysfunction and Post-Traumatic Stress Disorder in School Students during the Second COVID-19 Epidemic Wave in Peru. Int. J. Environ. Res. Public Health.

[12] Nguyen H.T.M., Nguyen H.V., Zouini B., Senhaji M., Bador K., Meszaros Z.S., Stevanovic D., Kerekes N. (2022). The COVID-19 Pandemic and Adolescents’ Psychological Distress: A Multinational Cross-Sectional Study. Int. J. Environ. Res. Public Health.

[13] Ponce V.V., Garrido M.V., Peralta C.I., Astudillo D., Malca J.T., Manrique E.O., Quispe E.T. (2020). Factores Asociados al Afrontamiento Psicológico Frente a La COVID-19 Durante El Periodo de Cuarentena. Rev. Cuba. Med. Mil..

[14] COVID-19 Pandemic Triggers 25% Increase in Prevalence of Anxiety and Depression Worldwide. https://www.who.int/news/item/02-03-2022-covid-19-pandemic-triggers-25-increase-in-prevalence-of-anxiety-and-depression-worldwide.

[15] Chutiyami M., Cheong A.M.Y., Salihu D., Bello U.M., Ndwiga D., Maharaj R., Naidoo K., Kolo M.A., Jacob P., Chhina N. (2021). COVID-19 Pandemic and Overall Mental Health of Healthcare Professionals Globally: A Meta-Review of Systematic Reviews. Front. Psychiatry.

[16] Schaughency K.C., Watkins E.Y., Preston S.L. (2021). Is Suicide a Social Phenomenon during the COVID-19 Pandemic? Differences by Birth Cohort on Suicide among Active Component Army Soldiers, 1 January 2000–4 June 2021. MSMR.

[17] Singh O.P. (2021). Comprehensive Mental Health Action Plan 2013–2030: We Must Rise to the Challenge. Indian J. Psychiatry.

[18] Comprehensive Mental Health Action Plan 2013–2030. https://www.who.int/publications-detail-redirect/9789240031029.

[19] Van Orden K.A., Witte T.K., Cukrowicz K.C., Braithwaite S., Selby E.A., Joiner T.E. (2010). The Interpersonal Theory of Suicide. Psychol. Rev..

[20] Patel V., Chisholm D., Parikh R., Charlson F.J., Degenhardt L., Dua T., Ferrari A.J., Hyman S., Laxminarayan R., Levin C. (2016). Addressing the Burden of Mental, Neurological, and Substance Use Disorders: Key Messages from Disease Control Priorities, 3rd Edition. Lancet.

[21] Bilsen J. (2018). Suicide and Youth: Risk Factors. Front. Psychiatry.

[22] Shen H., Gelaye B., Huang H., Rondon M.B., Sanchez S., Duncan L.E. (2020). Polygenic Prediction and GWAS of Depression, PTSD, and Suicidal Ideation/Self-Harm in a Peruvian Cohort. Neuropsychopharmacology.

[23] Maciejewski D.F., Renteria M.E., Abdellaoui A., Medland S.E., Few L.R., Gordon S.D., Madden P.A.F., Montgomery G., Trull T.J., Heath A.C. (2017). The Association of Genetic Predisposition to Depressive Symptoms with Non-Suicidal and Suicidal Self-Injuries. Behav. Genet..

[24] Gunnell D., Appleby L., Arensman E., Hawton K., John A., Kapur N., Khan M., O’Connor R.C., Pirkis J., Appleby L. (2020). Suicide Risk and Prevention during the COVID-19 Pandemic. Lancet Psychiatry.

[25] López S.M.A., Bedoya Mejía S., Henao Valencia M.C., Velasquez Correa J.C., Grisales Romero H. (2020). Prevalencia de depresión en soldados regulares de un batallón de una ciudad colombiana, 2017. Rev. Médica Risaralda.

[26] Franklin J.C., Ribeiro J.D., Fox K.R., Bentley K.H., Kleiman E.M., Huang X., Musacchio K.M., Jaroszewski A.C., Chang B.P., Nock M.K. (2017). Risk Factors for Suicidal Thoughts and Behaviors: A Meta-Analysis of 50 Years of Research. Psychol. Bull..

[27] Tsegay A., Damte A., Kiros A. (2020). Determinants of Suicidal Ideation among Patients with Mental Disorders Visiting Psychiatry Outpatient Unit in Mekelle Town, Psychiatric Clinics, Tigray, Northern Ethiopia: A Case–Control Study. Ann. Gen. Psychiatry.

[28] Vargas I., Perlis M.L., Grandner M., Gencarelli A., Khader W., Zandberg L.J., Klingaman E.A., Goldschmied J.R., Gehrman P.R., Brown G.K. (2020). Insomnia Symptoms and Suicide-Related Ideation in U.S. Army Service Members. Behav. Sleep Med..

[29] Lin H.-T., Lai C.-H., Perng H.-J., Chung C.-H., Wang C.-C., Chen W.-L., Chien W.-C. (2018). Insomnia as an Independent Predictor of Suicide Attempts: A Nationwide Population-Based Retrospective Cohort Study. BMC Psychiatry.

[30] Tubbs A.S., Fernandez F.-X., Perlis M.L., Hale L., Branas C.C., Barrett M., Chakravorty S., Khader W., Grandner M.A. (2021). Suicidal Ideation Is Associated with Nighttime Wakefulness in a Community Sample. Sleep.

[31] Ortiz-Calvo E., Martínez-Alés G., Mediavilla R., González-Gómez E., Fernández-Jiménez E., Bravo-Ortiz M.-F., Moreno-Küstner B. (2022). The Role of Social Support and Resilience in the Mental Health Impact of the COVID-19 Pandemic among Healthcare Workers in Spain. J. Psychiatr. Res..

[32] Bentley K.H., Franklin J.C., Ribeiro J.D., Kleiman E.M., Fox K.R., Nock M.K. (2016). Anxiety and Its Disorders as Risk Factors for Suicidal Thoughts and Behaviors: A Meta-Analytic Review. Clin. Psychol. Rev..

[33] Ilgen M.A., Bohnert A.S.B., Ignacio R.V., McCarthy J.F., Valenstein M.M., Kim H.M., Blow F.C. (2010). Psychiatric Diagnoses and Risk of Suicide in Veterans. Arch. Gen. Psychiatry.

[34] Gutarra Condor B.A. (2019). Estructura Familiar y Riesgo Suicida Del Personal Voluntario en Servicio Militar—Cuartel 9 de Diciembre, Huancayo, 2018.

[35] Díaz-Vélez C., Failoc-Rojas V.E., Valladares-Garrido M.J., Colchado J., Carrera-Acosta L., Becerra M., Moreno Paico D., Ocampo-Salazar E.T. (2021). SARS-CoV-2 Seroprevalence Study in Lambayeque, Peru. June-July 2020. PeerJ.

[36] Lambayeque: Más de mil miembros del Ejército serán vacunados contra el COVID-19. https://elcomercio.pe/peru/lambayeque-mas-de-mil-miembros-del-ejercito-seran-vacunados-contra-el-covid-19-nnpp-noticia/.

[37] Harris P.A., Taylor R., Thielke R., Payne J., Gonzalez N., Conde J.G. (2009). Research Electronic Data Capture (REDCap)—A Metadata-Driven Methodology and Workflow Process for Providing Translational Research Informatics Support. J. Biomed. Inform..

[38] Harris P.A., Taylor R., Minor B.L., Elliott V., Fernandez M., O’Neal L., McLeod L., Delacqua G., Delacqua F., Kirby J. (2019). The REDCap Consortium: Building an International Community of Software Platform Partners. J. Biomed. Inform..

[39] Campas M.A., Santoyo F. (2019). Psychometric Properties of the Plutchik Suicide Risk Scale in a Sample of Young Mexicans Deprived of Liberty. LA Ref..

[40] Torres L.C. (2021). Validation and Standardization of the Plutchik Suicide Risk Scale in the Civil Population and Active Police in Colombia. Gac. Méd. Caracas.

[41] Suárez-Colorado Y., Palacio-Sañudo J., Caballero-Domínguez C.C., Pineda-Roa C.A. (2019). Adaptación, validez de constructo y confiabilidad de la escala de riesgo suicida Plutchik en adolescentes colombianos. Rev. Latinoam. Psicol..

[42] García-León M.-Á., González-Gómez A., Robles-Ortega H., Padilla J.-L., Peralta-Ramírez M.-I., García-León M.-Á., González-Gómez A., Robles-Ortega H., Padilla J.-L., Peralta-Ramírez M.-I. (2019). Propiedades Psicométricas de La Escala de Resiliencia de Connor y Davidson (CD-RISC) En Población Española. An. Psicol..

[43] Leiva León N.F. (2021). La resilencia como factor asociado al Sindrome de Burnout, depresión y ansiedad en el personal de salud que labora en las Unidades de Cuidados Intensivos durante la pandemia COVID-19 en el Perú. DSpace Repos..

[44] Karaırmak O. (2010). Establishing the Psychometric Qualities of the Connor-Davidson Resilience Scale (CD-RISC) Using Exploratory and Confirmatory Factor Analysis in a Trauma Survivor Sample. Psychiatry Res..

[45] Spitzer R.L., Kroenke K., Williams J.B.W., Löwe B. (2006). A Brief Measure for Assessing Generalized Anxiety Disorder: The GAD-7. Arch. Intern. Med..

[46] García-Campayo J., Zamorano E., Ruiz M.A., Pérez-Páramo M., López-Gómez V., Rejas J. (2012). The Assessment of Generalized Anxiety Disorder: Psychometric Validation of the Spanish Version of the Self-Administered GAD-2 Scale in Daily Medical Practice. Health Qual. Life Outcomes.

[47] Calderón M., Gálvez-Buccollini J.A., Cueva G., Ordoñez C., Bromley C., Fiestas F. (2012). Validación de La Versión Peruana Del PHQ-9 Para El Diagnóstico de Depresión. Rev. Peru. Med. Exp. Salud Publica.

[48] Baader M.T., Molina F.J.L., Venezian B.S., Rojas C.C., Farías S.R., Fierro-Freixenet C., Backenstrass M., Mundt C. (2012). Validación y Utilidad de La Encuesta PHQ-9 (Patient Health Questionnaire) En El Diagnóstico de Depresión En Pacientes Usuarios de Atención Primaria En Chile. Rev. Chil. Neuro-Psiquiatr..

[49] Flores Morales R., Reyes Pérez V., Reidl Martínez L.M. (2012). Síntomas De Estrés Postraumático (Ept) En Periodistas Mexicanos Que Cubren La Guerra Contra El Narcotráfico. Suma Psicológica.

[50] Durón-Figueroa R., Cárdenas-López G., Castro-Calvo J., Rosa-Gómez A.D., la Durón-Figueroa R., Cárdenas-López G., Castro-Calvo J., Rosa-Gómez A.D. (2019). la Adaptación de la Lista Checable de Trastorno por Estrés Postraumático para DSM-5 en Población Mexicana. Acta Investig. Psicológica.

[51] González-Rodríguez R., Domínguez Alonso J., Verde-Diego C., Frieiro Padín P. (2020). Psychometric Properties of the Maslach Burnout Inventory—Human Services in Social Work Professionals in Spain. Health Soc. Care Community.

[52] Olivares-Faúndez V.E., Mena-Miranda L., Jélvez-Wilker C., Macía-Sepúlveda F. (2014). Validez factorial del Maslach Burnout Inventory Human Services (MBI-HSS) en profesionales chilenos. Univ. Psychol..

[53] Sierra J.C., Guillén-Serrano V., Santos-Iglesias P. (2008). Insomnia Severity Index: Some indicators about its reliability and validity on an older adults sample. Rev. Neurol..

[54] Fernandez-Mendoza J., Rodriguez-Muñoz A., Vela-Bueno A., Olavarrieta-Bernardino S., Calhoun S.L., Bixler E.O., Vgontzas A.N. (2012). The Spanish Version of the Insomnia Severity Index: A Confirmatory Factor Analysis. Sleep Med..

[55] Mantilla Toloza S.C., Gómez-Conesa A. (2007). El Cuestionario Internacional de Actividad Física. Un instrumento adecuado en el seguimiento de la actividad física poblacional. Rev. Iberoam. Fisioter. Kinesiol..

[56] Limb E.S., Ahmad S., Cook D.G., Kerry S.M., Ekelund U., Whincup P.H., Victor C.R., Iliffe S., Ussher M., Fox-Rushby J. (2019). Measuring Change in Trials of Physical Activity Interventions: A Comparison of Self-Report Questionnaire and Accelerometry within the PACE-UP Trial. Int. J. Behav. Nutr. Phys. Act..

[57] Ahorsu D.K., Lin C.-Y., Imani V., Saffari M., Griffiths M.D., Pakpour A.H. (2020). The Fear of COVID-19 Scale: Development and Initial Validation. Int. J. Ment. Health Addict..

[58] Huarcaya-Victoria J., Villarreal-Zegarra D., Podestà A., Luna-Cuadros M.A. (2022). Psychometric Properties of a Spanish Version of the Fear of COVID-19 Scale in General Population of Lima, Peru. Int. J. Ment. Health Addict..

[59] Nikopoulou V.A., Holeva V., Parlapani E., Karamouzi P., Voitsidis P., Porfyri G.N., Blekas A., Papigkioti K., Patsiala S., Diakogiannis I. (2022). Mental Health Screening for COVID-19: A Proposed Cutoff Score for the Greek Version of the Fear of COVID-19 Scale (FCV-19S). Int. J. Ment. Health Addict..

[60] Ursano R.J., Heeringa S.G., Stein M.B., Jain S., Raman R., Sun X., Chiu W.T., Colpe L.J., Fullerton C.S., Gilman S.E. (2015). Prevalence and Correlates of Suicidal Behavior among New Soldiers in the U.S. Army: Results from the Army Study to Assess Risk and Resilience in Servicemembers (Army STARRS). Depress. Anxiety.

[61] Kopacz M.S., Currier J.M., Drescher K.D., Pigeon W.R. (2016). Suicidal Behavior and Spiritual Functioning in a Sample of Veterans Diagnosed with PTSD. J. Inj. Violence Res..

[62] Roberge E.M., Harris J.A., Weinstein H.R., Rozek D.C. (2021). Treating Veterans at Risk for Suicide: An Examination of the Safety, Tolerability, and Outcomes of Cognitive Processing Therapy. J. Trauma. Stress.

[63] Gebremeskel T.G., Berhe M., Tesfa Berhe E. (2022). Suicide Attempts Among Adult Eritrean Refugees in Tigray, Ethiopia: Prevalence and Associated Factors. Risk Manag. Healthc Policy.

[64] Pavez P., Santander N., Carranza J., Vera-Villarroel P. (2009). Factores de Riesgo Familiares Asociados a La Conducta Suicida En Adolescentes Con Trastorno Depresivo. Revista Médica Chile.

[65] Harmer B., Lee S., Duong T., Vi H., Saadabadi A. (2022). Suicidal Ideation. StatPearls.

[66] Fogle B.M., Tsai J., Mota N., Harpaz-Rotem I., Krystal J.H., Southwick S.M., Pietrzak R.H. (2020). The National Health and Resilience in Veterans Study: A Narrative Review and Future Directions. Front. Psychiatry.

[67] Kessler R.C., Heeringa S.G., Stein M.B., Colpe L.J., Fullerton C.S., Hwang I., Naifeh J.A., Nock M.K., Petukhova M., Sampson N.A. (2014). Thirty-Day Prevalence of DSM-IV Mental Disorders Among Nondeployed Soldiers in the US Army. JAMA Psychiatry.

[68] García-Rivera B.R., García-Alcaraz J.L., Mendoza-Martínez I.A., Olguin-Tiznado J.E., García-Alcaráz P., Aranibar M.F., Camargo-Wilson C. (2021). Influence of COVID-19 Pandemic Uncertainty in Negative Emotional States and Resilience as Mediators against Suicide Ideation, Drug Addiction and Alcoholism. Int. J. Environ. Res. Public Health.

[69] Conner K.R., McCarthy M.D., Bajorska A., Caine E.D., Tu X.M., Knox K.L. (2012). Mood, Anxiety, and Substance-Use Disorders and Suicide Risk in a Military Population Cohort. Suicide Life Threat. Behav..

[70] Byrne S.P., Fogle B.M., Asch R., Esterlis I., Harpaz-Rotem I., Tsai J., Pietrzak R.H. (2021). The Hidden Burden of Social Anxiety Disorder in U.S. Military Veterans: Results from the National Health and Resilience in Veterans Study. J. Affect. Disord..

[71] Yin Q., Dong W., Chen A., Song X., Hou T., Cai W., Deng G. (2020). The Influence of Social Anxiety on Interpersonal Information Processing in a Military-Life Environment: A Cross-Sectional Study. Medicine.

[72] Moradi Y., Dowran B., Sepandi M. (2021). The Global Prevalence of Depression, Suicide Ideation, and Attempts in the Military Forces: A Systematic Review and Meta-Analysis of Cross Sectional Studies. BMC Psychiatry.

[73] Jimenez F., Dominguez Espinosa A. (2010). Influencia de La Deseabilidad Social (DS) En Reportes de Capacitación. Psicol. Iberoam..

[74] Sánchez-Teruel D., Muela-Martínez J.-A., González-Cabrera M., Herrera M.-R.F.-A.Y., García-León A. (2018). Variables relacionadas con la tentativa suicida en una provincia de España durante tres años (2009-2011). Ciênc. Saúde Colet..

[75] Borges G., Nock M.K., Haro Abad J.M., Hwang I., Sampson N.A., Alonso J., Andrade L.H., Angermeyer M.C., Beautrais A., Bromet E. (2010). Twelve-Month Prevalence of and Risk Factors for Suicide Attempts in the World Health Organization World Mental Health Surveys. J. Clin. Psychiatry.

